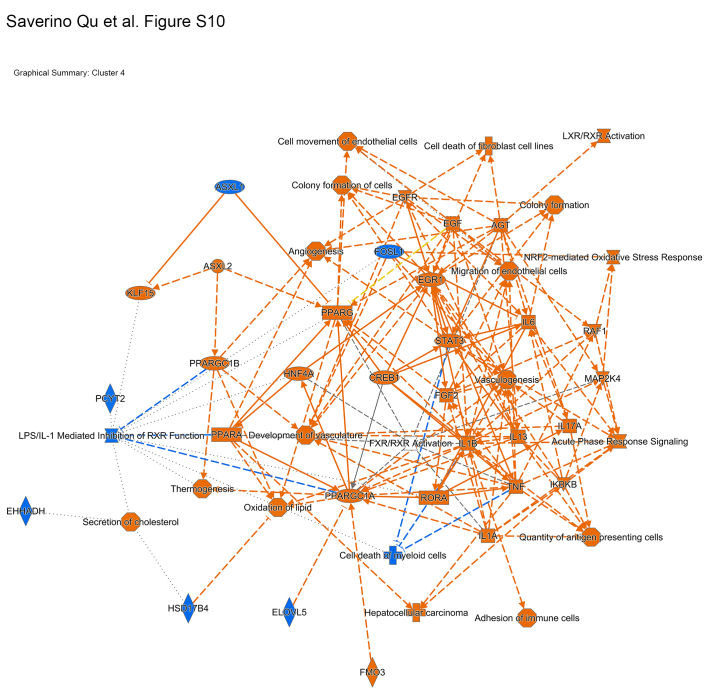# Spatial transcriptomics unravels palmitoylation and zonation-dependent gene regulation by AEG-1 in mouse liver

**DOI:** 10.1016/j.jbc.2024.107322

**Published:** 2024-04-25

**Authors:** Alissa Saverino, Xufeng Qu, Rachel G. Mendoza, Suchismita Raha, Debashri Manna, Ali Gawi Ermi, Mark A. Subler, Jolene J. Windle, Jinze Liu, Devanand Sarkar

**Affiliations:** 1 Department of Human and Molecular Genetics, Virginia Commonwealth University, Richmond, Virginia, USA; 2 Massey Comprehensive Cancer Center, Virginia Commonwealth University, Richmond, Virginia, USA; 3 Department of Biostatistics, Virginia Commonwealth University, Richmond, Virginia, USA; 4 VCU Institute of Molecular Medicine, Virginia Commonwealth University, Richmond, Virginia, USA

**Keywords:** AEG-1, mouse liver, zonation, spatial transcriptomics, inflammation

## Abstract

Obesity-induced metabolic dysfunction-associated steatohepatitis (MASH) leads to hepatocellular carcinoma (HCC). Astrocyte-elevated gene-1/Metadherin (AEG-1/MTDH) plays a key role in promoting MASH and HCC. AEG-1 is palmitoylated at residue cysteine 75 (Cys75) and a knock-in mouse representing mutated Cys75 to serine (AEG-1-C75S) showed activation of MASH- and HCC-promoting gene signature when compared to wild-type littermates (AEG-1-WT). The liver consists of three zones, periportal, mid-lobular, and pericentral, and zone-specific dysregulated gene expression impairs metabolic homeostasis in the liver, contributing to MASH and HCC. Here, to elucidate how palmitoylation influences AEG-1-mediated gene regulation in regard to hepatic zonation, we performed spatial transcriptomics (ST) in the livers of AEG-1-WT and AEG-1-C75S littermates. ST identified six different clusters in livers and using zone- and cell-type-specific markers we attributed specific zones and cell types to specific clusters. Ingenuity Pathway Analysis (IPA) of differentially expressed genes in each cluster unraveled activation of pro-inflammatory and MASH- and HCC-promoting pathways, mainly in periportal and pericentral hepatocytes, in AEG-1-C75S liver compared to AEG-1-WT. Interestingly, in AEG-1-C75S liver, the mid-lobular zone exhibited widespread inhibition of xenobiotic metabolism pathways and inhibition of PXR/RXR and LXR/RXR activation, *versus* AEG-1-WT. In conclusion, AEG-1-C75S mutant exhibited zone-specific differential gene expression, which might contribute to metabolic dysfunction and dysregulated drug metabolism leading to MASH and HCC.

Hepatocellular carcinoma (HCC) is the most common form of primary liver cancer and poses a global health burden due to its increasing incidence and mortality rates (1). This is especially related to HCC’s prominent link to obesity-associated metabolic dysfunction-associated steatohepatitis (MASH, previously known as NASH), a subtype of metabolic dysfunction-associated fatty liver disease (MAFLD, previously known as NAFLD) (2). MAFLD and its progressive variant MASH are known to inflict substantial disruptions on liver metabolism, making them fundamental factors in HCC occurrence and development (2). Imbalances in these metabolic functions underscore the significance of proper spatial organization across this organ. Therefore, research efforts aimed at examining the metabolic activity in MASH- and HCC-affected livers are crucial to uncovering the molecular mechanisms that prompt HCC pathogenesis.

The liver consists of three established zones—zone 1 (periportal), zone 2 (mid-lobular), and zone 3 (pericentral). Each zone bears the responsibility for carrying out specific metabolic functions, determined by the cells’ relative localization and, ultimately, which zone they reside in (Fig. 1) (3). The separation of these processes is principally controlled by differential gene expression between the cells characterizing each zone, as well as the genes involved in these cellular functions. This zonal segregation is vital to ensuring the efficient execution of metabolic tasks within the liver. Dysregulation of the spatial and temporal gene expression patterns appears to be intrinsically linked to the onset and progression of liver diseases, namely, HCC and MASH (4). Thus, the importance of liver spatial organization relates to the proper assignment of metabolic tasks to their respective zonation.

**Figure 1:**
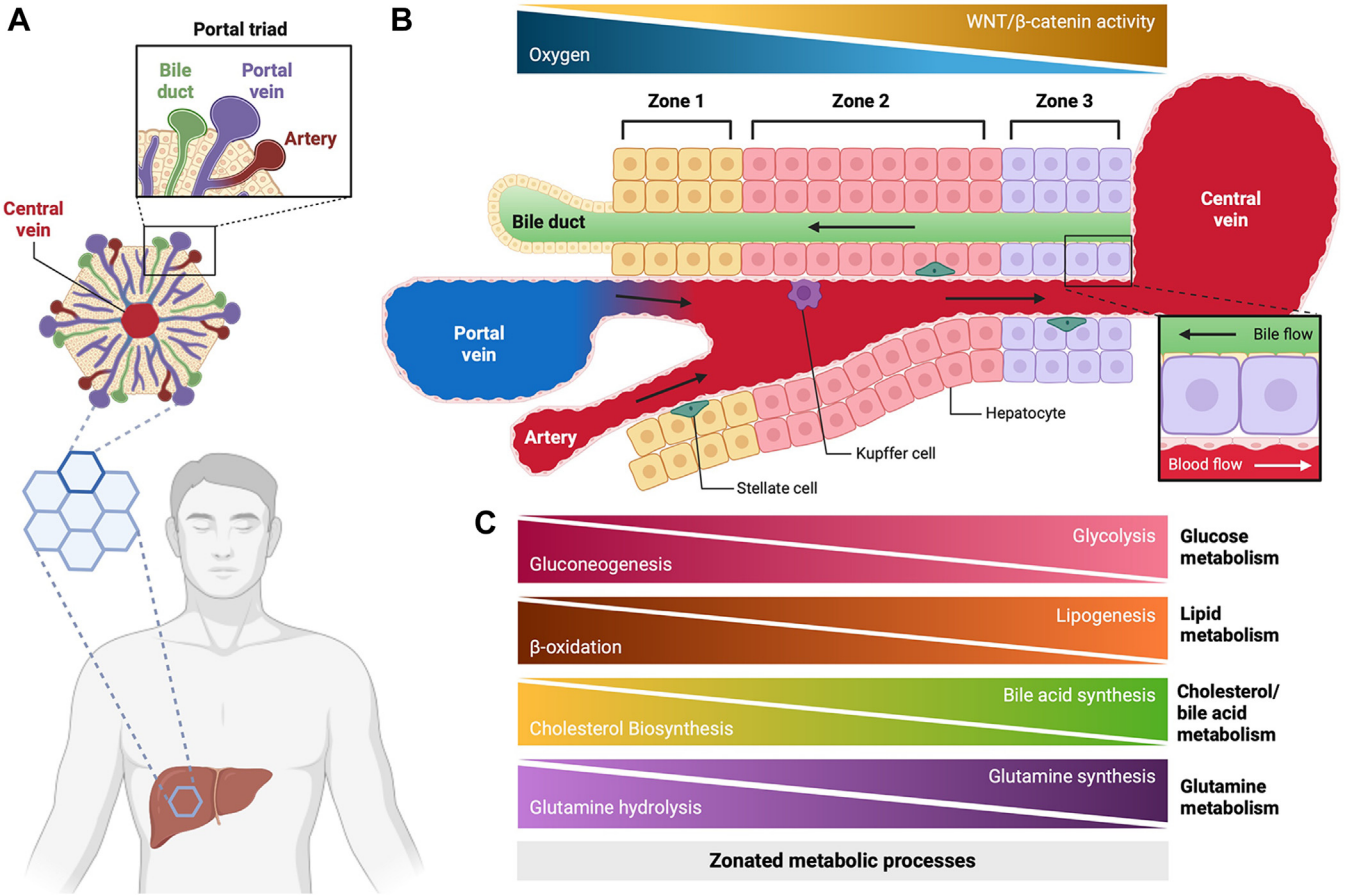
**Cartoons showing liver zonation.***A*, diagram of hexagonal units in the liver with a central vein at the center and portal triad composed of bile duct, portal vein and hepatic artery at the periphery. *B*, diagram of distribution of the three zones of hepatocytes related to their position to central vein or portal triad. *C*, metabolic functions carried out by hepatocytes in each zone.

Astrocyte elevated gene-1 (AEG-1, also known as metadherin or MTDH) functions as an oncogene and plays a seminal role in promoting MASH and HCC as described in our previous studies (5, 6, 7, 8, 9, 10). AEG-1 is a scaffold protein and exerts its functions by protein-protein and protein-RNA interactions (11). As an oncogene AEG-1 overexpression contributes to all hallmarks of cancer (11). MASH has two major components, steatosis and inflammation. AEG-1 promotes steatosis by inhibiting peroxisome proliferator-activated receptor alpha (PPARα), a master regulator of fatty acid β-oxidation, and translationally upregulating fatty acid synthesizing enzymes, notably fatty acid synthase (12). AEG-1 functions as a scaffold in multiple steps in the activation process of NF-κB, a master regulator of inflammatory cytokines, and by activating NF-κB AEG-1 promotes inflammation (13, 14, 15). AEG-1 is a 582 amino acid protein that has a single cysteine at position 75 (Cys75) and we recently demonstrated that AEG-1 is palmitoylated at Cys75 (16). Palmitoylation, a post-translational modification in which fatty acids are covalently attached to cysteine residues, is largely recognized as a positive regulator of protein function (17). We interrogated how palmitoylation regulates AEG-1 function by generating a knock-in mouse *via* CRISPR/Cas9 where Cys75 was mutated to serine (AEG-1-C75S) (16). RNA-sequencing analysis of AEG-1-WT and AEG-C75S hepatocytes unraveled that palmitoylation negatively regulates AEG-1 function such that genes regulating steatosis, inflammation, and tumorigenesis are significantly more abundant in AEG-C75S hepatocytes compared to AEG-1-WT (16).

In this study, we delved into the nuanced interplay of spatial organization, metabolic disruption, and AEG-1-mediated gene regulation within murine livers. We seek to bridge the gap between spatial transcriptomics (ST), liver zonation and metabolic organization, and AEG-1 palmitoylation to further elucidate the molecular mechanisms underlying MASH and HCC pathogenesis. By utilizing ST technology to investigate tissue-wide gene expression patterns, we aim to shed light on the spatial organization of distinct cell populations and the zonation of metabolic activity across the tissue. These findings, coupled with insights into AEG-1-mediated regulation, can offer novel perspectives on HCC etiology and open avenues for the development of targeted therapies for HCC.

## Results

### Spatial transcriptomics reveals distinct clusters and implicates liver zonation

**Figure 2:**
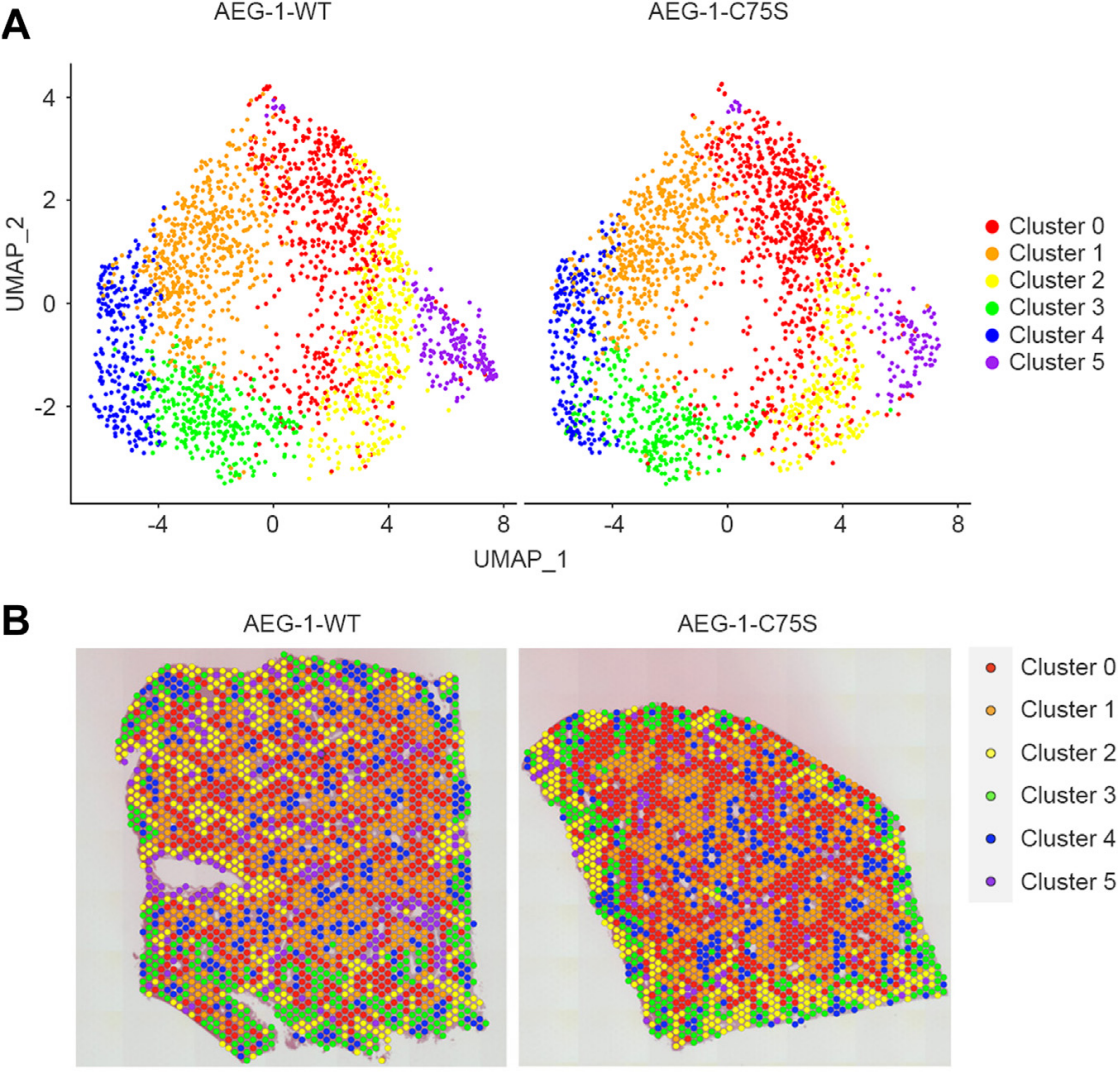
**ST identified six different clusters in mouse livers.***A*, uniform manifold approximation and projection (UMAP) plots of six clusters in AEG-1-WT and AEG-1-C75S livers. *B*, distribution of six different clusters in AEG-1-WT and AEG-1-C75S livers identified by ST analysis.

**Supporting Figure S1:**
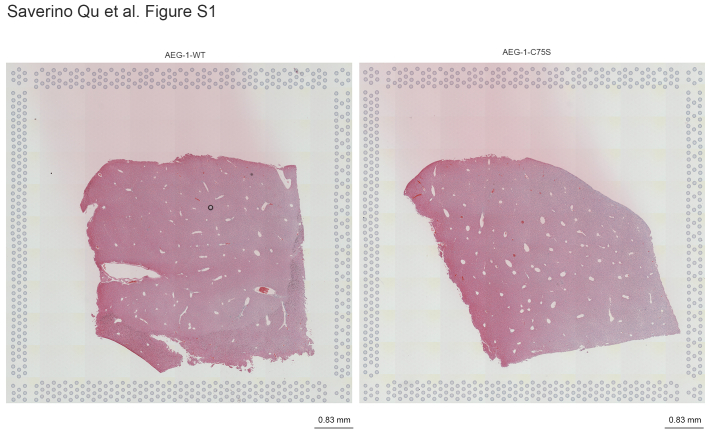


ST was first employed to analyze gene expression in various liver regions. H&E-stained slides of liver sections are shown in Fig. S1. For each sample, spots with counts greater than 50,000, and feature numbers less than 100 or greater than 7000 were removed. After this quality control, for AEG-1-WT and AEG-1-C75S livers, the number of spots was 2309 and 2163, respectively, and median genes per spot were 2316 and 2167, respectively, indicating consistency between the two samples. In each sample, we identified six distinct clusters of spatial spots, each characterized by unique gene expression profiles (Fig. 2). A list of highly expressed genes to annotate each cluster is presented in Table S1. These clusters signify the complexity of mouse liver tissue and its intricate cellular composition, as each cluster may correspond to a specific cell type and/or zone within the liver. Therefore, the concept of zonation, a key aspect of liver architecture, is likely quite pertinent to our findings. The presence of these distinct clusters in our samples suggests that the zonation may be driven, at least in part, by the presence and distribution of different cell types with unique gene expression profiles. To accurately characterize these clusters, we integrated the expression data for each individual cluster with known expression markers representative of a particular zone or cell type.

### Zone-specific marker analysis locates periportal, mid-lobular, and pericentral zones

**Figure 3:**
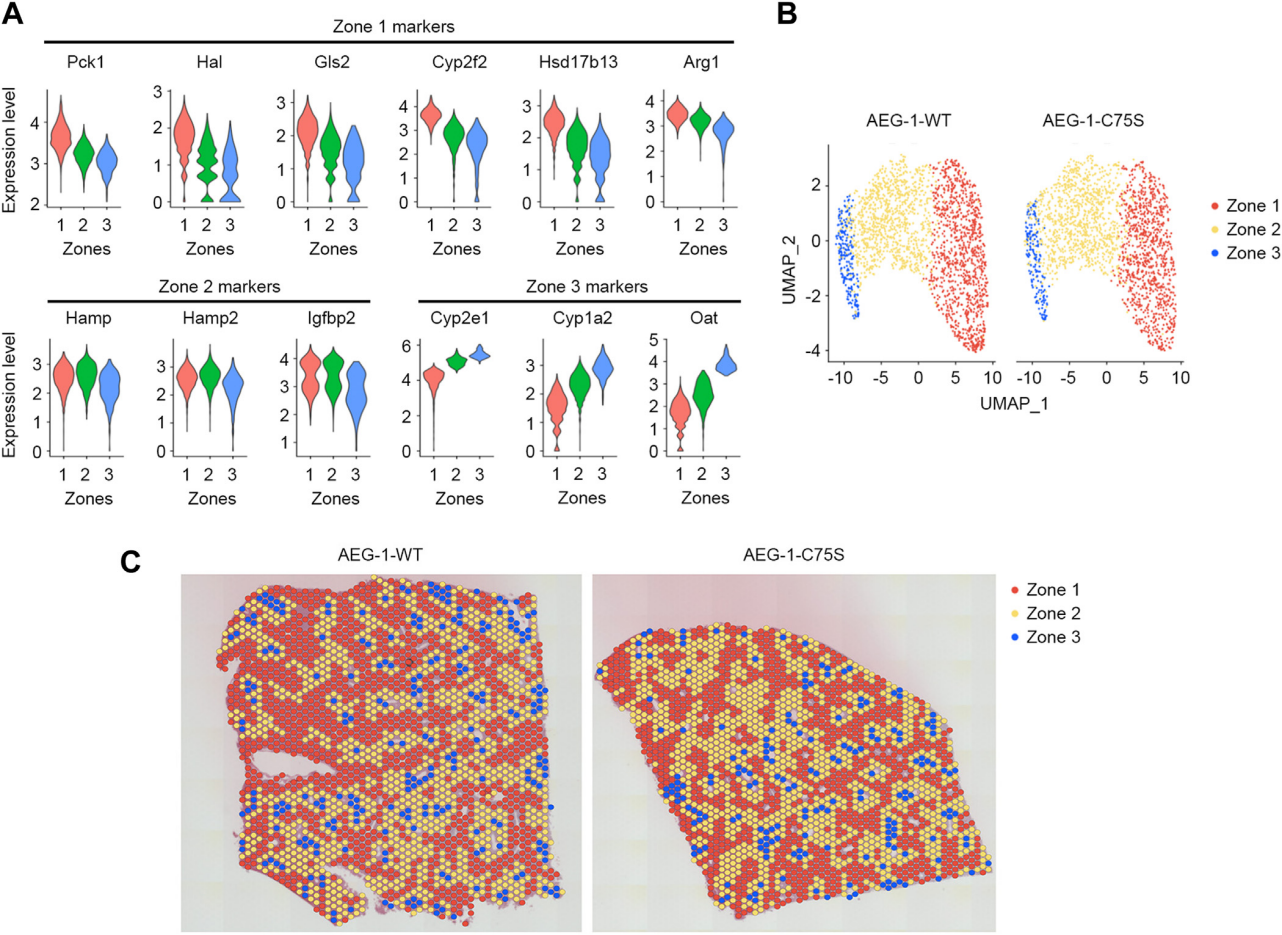
**Hepatocytes are distributed in three distinct zones in AEG-1-WT and AEG-1-C75S livers.***A*, violin plots of zone-specific gene markers in different zones of WT mouse livers. *B*, uniform manifold approximation and projection (UMAP) plots of three zones of AEG-1-WT and AEG-1-C75S livers. *C*, distribution of zone-specific hepatocytes in AEG-1-WT and AEG-1-C75S livers based on the expression of zone-specific gene markers.

We utilized the gene expression data generated by the spatial analysis to determine which areas of mouse liver samples represent each zone. Markers for zone 1 (periportal) hepatocytes (*Pck1, Hal, Gls2, Cyp2f2, Hsd17b13, Ass1, Arg1*), zone 2 (mid-lobular) hepatocytes (*Hamp, Hamp2, Igfbp2*), and zone 3 (pericentral) hepatocytes (*Cyp2e1, Cyp1a2, Oat*) are known to sufficiently characterize hepatic zonation and thus were employed in our targeted clustering analysis (3). The spatial spots were re-clustered using the gene expression profiles of the 13 zone-specific markers. This allows us to identify the three established liver zones, each of which is enriched with distinct markers, as shown in the violin plots of zone-specific markers (Fig. 3*A*). The hepatocytes in each zone were similarly distributed between AEG-1-WT and AEG-1-C75S livers without showing significant differences in their numbers and any overlap (Fig. 3, *B* and *C*). These distinct patterns of expression within the periportal, mid-lobular, and pericentral hepatocytes provided a visual representation of where the zones are localized in our mouse tissue samples.

### Localization of distinct non-parenchymal cell types in liver tissue *via* clustering analysis

**Figure 4:**
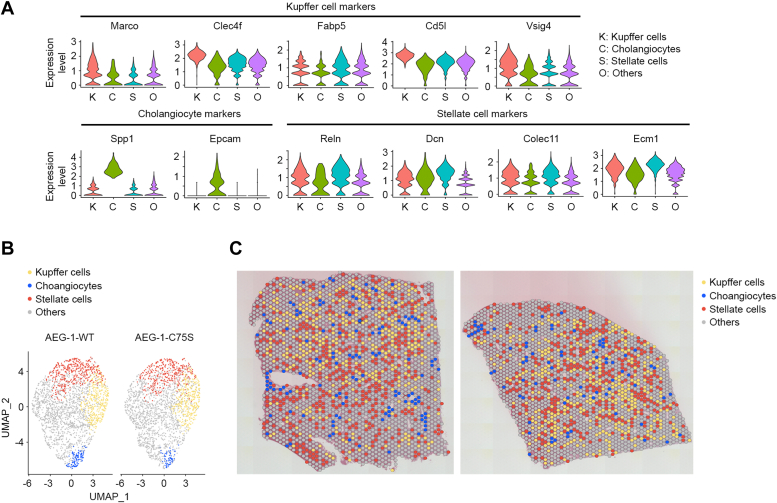
**Non-parenchymal cells are distributed evenly in AEG-1-WT and AEG-1-C75S livers.***A*, violin plots of indicated cell type-specific gene markers in different cell types of WT mouse livers. *B*, uniform manifold approximation and projection (UMAP) plots of different cell types of AEG-1-WT and AEG-1-C75S livers. Other indicates predominantly hepatocytes. *C*, distribution of principal non-parenchymal cells in AEG-1-WT and AEG-1-C75S livers based on expression of cell-type specific gene markers.

After examining liver zonation, we subsequently aimed to localize principal non-parenchymal cell types. Cell type-specific markers for Kupffer cells (*Marco, Clec4f, Fabp5, Cd5l, Vsig4*), cholangiocytes (*Spp1, Epcam*), and hepatic stellate cells (*Reln, Dcn, Colec11, Ecm1*) were utilized to determine areas of enriched spots. The violin plot of these cell type-specific markers demonstrated their expression in each specific cell type (Fig. 4*A*). The non-parenchymal cells were similarly distributed between AEG-1-WT and AEG-1-C75S livers without showing significant differences in their numbers (Fig. 4*B*). We observed nearly exclusive expression patterns of each cell type’s markers in AEG-1-WT and AEG-1-C75S livers (Fig. 4*C*).

**Figure 5:**
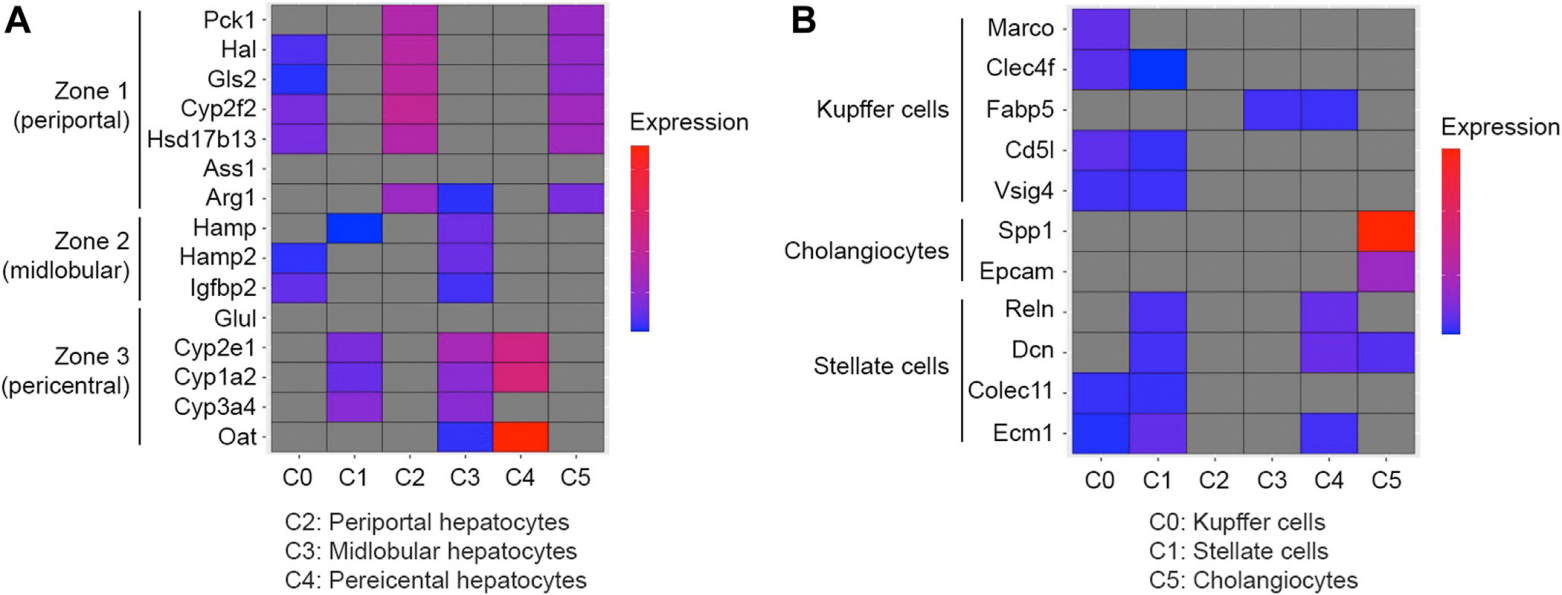
**Markers identify zones and cell types in the clusters.** Heatmap showing markers of different zones (*A*) and different non-parenchymal cell types (*B*) in six different clusters.

We checked the expression of the hepatocyte zone-specific markers in the six different clusters (Fig. 5*A*). Cluster two exclusively expressed zone 1-specific markers, but not markers for zones two or 3, indicating that this cluster contains predominantly zone 1 (periportal) hepatocytes. Cluster three expressed all the markers of zone 2 as well as markers of zone 3, suggesting that this cluster predominantly represents zone 2 (mid-lobular) hepatocytes. Cluster four expressed only zone 3 markers, but did not express markers for zones 1 and 2, indicating that this cluster contains zone 3 (pericentral) hepatocytes. Cluster 0 expressed markers of both periportal and mid-lobular zones suggesting that this cluster represents mid-lobular hepatocytes close to the periportal zone. Cluster one expressed markers of both pericentral and mid-lobular zones suggesting that this cluster represents mid-lobular hepatocytes close to the pericentral zone. Cluster five expressed markers of periportal hepatocytes but this is the only cluster expressing high levels of cholangiocyte marker Spp1, suggesting that this cluster predominantly contains cholangiocytes (Fig. 5*B*).

Similarly, we checked the expression of Kupffer cells-, cholangiocytes- and stellate cells-specific markers in the six different clusters (Fig. 5*B*). Cluster 0 expressed four out of 5 markers for macrophages and two out of 4 markers of stellate cells indicating that this cluster is enriched with predominantly macrophages along with stellate cells. Cluster 1 expressed three out of 5 markers for macrophages and all of the markers of stellate cells indicating that this cluster is enriched with predominantly stellate cells along with macrophages. Cluster 2 did not show expression of any of the non-parenchymal cells. This is the cluster that showed exclusive expression of zone 1 (periportal) markers indicating that this cluster is enriched with periportal hepatocytes. Similarly, Cluster 3 only expressed one marker for macrophages and this is the cluster that showed expression of all the markers of zone 2 (mid-lobular) and zone 3 (pericentral) hepatocytes, again indicating this cluster is also enriched with hepatocytes. Cluster four predominantly expressed markers of stellate cells thus displaying enrichment of these cells. Cluster 5 is the only cluster that expressed both cholangiocytes markers along with periportal hepatocytes indicating that this is the only cluster enriched with cholangiocytes and periportal hepatocytes.

### Activation of oncogenic, inflammatory, and fibrotic pathways in periportal and pericentral hepatocytes

**Figure 6:**
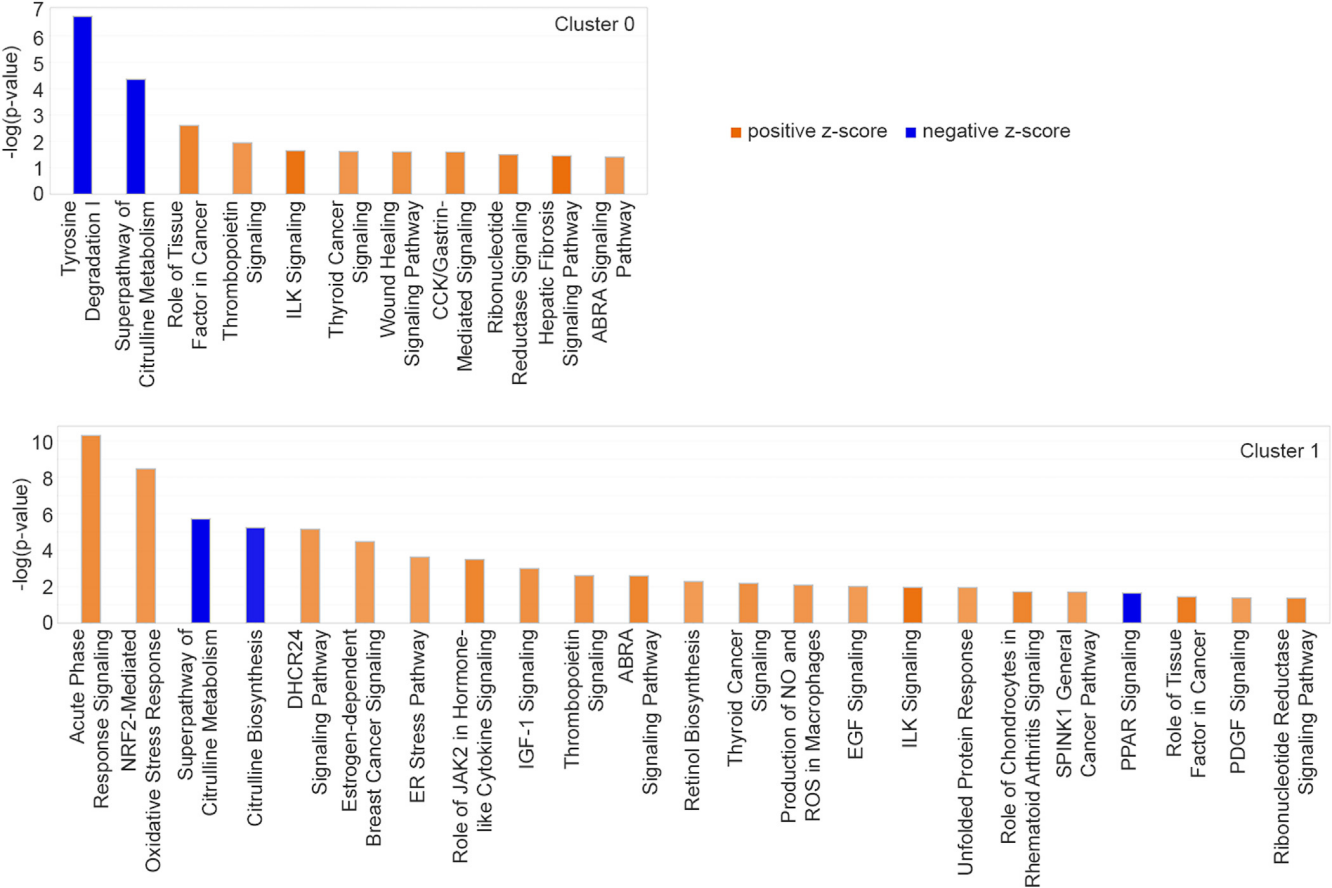
**Canonical pathways activated or inhibited in clusters 0 and one in AEG-1-C75S liver compared to AEG-1-WT.***Blue* indicates a negative z-score (*i.e.*, inhibition), *orange* indicates a positive z-score (*i.e.*, activation). Pathways are listed in order of *p*-values relating to how confident IPA is in the pathway’s relevance to gene expression results.

**Figure 7:**
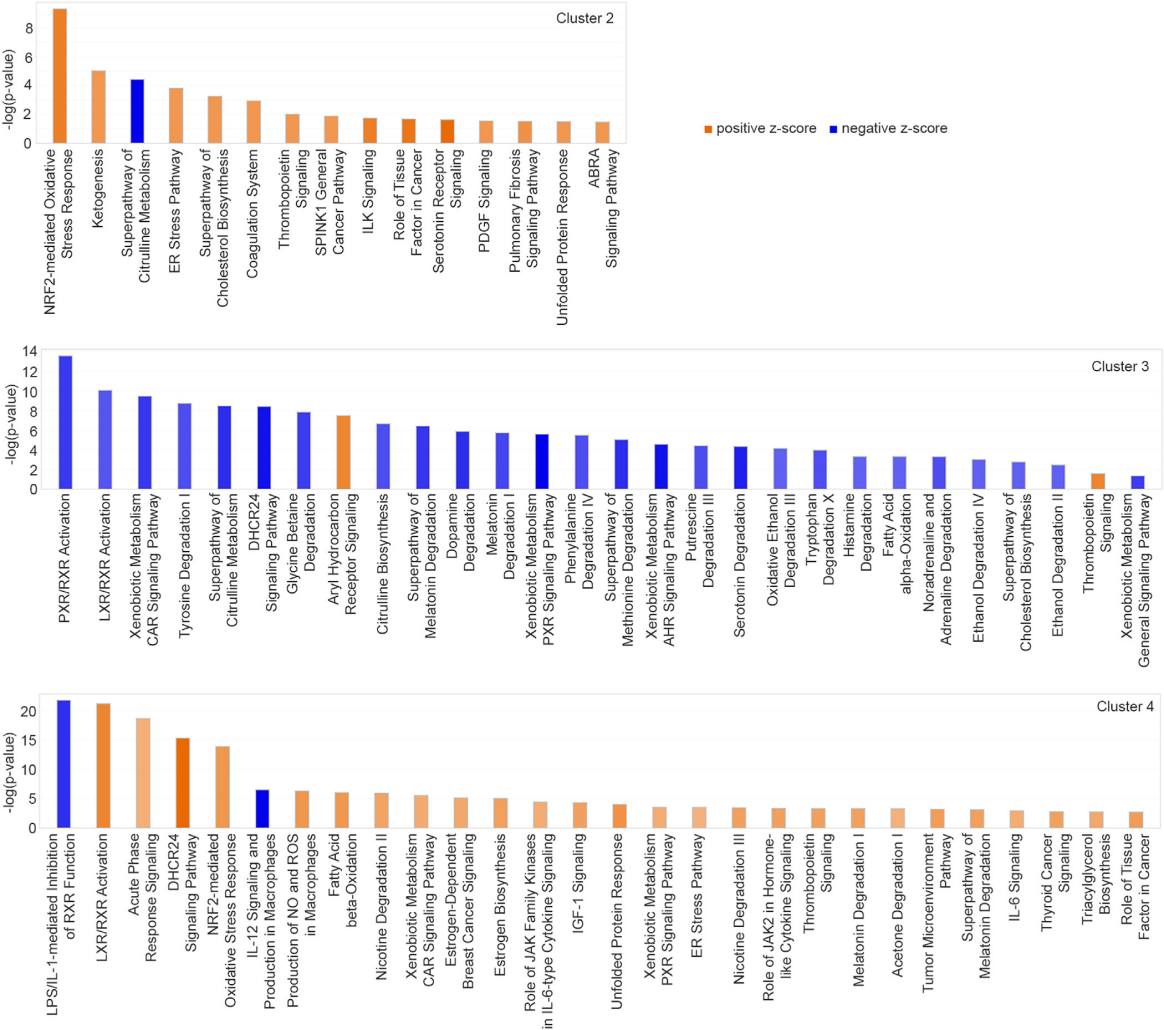
**Canonical pathways activated or inhibited in clusters 2, three and four in AEG-1-C75S liver compared to AEG-1-WT.***Blue* indicates a negative z-score (*i.e.*, inhibition), *orange* indicates a positive z-score (*i.e.*, activation). Pathways are listed in order of *p*-values relating to how confident IPA is in the pathway’s relevance to gene expression results.

**Figure 8:**
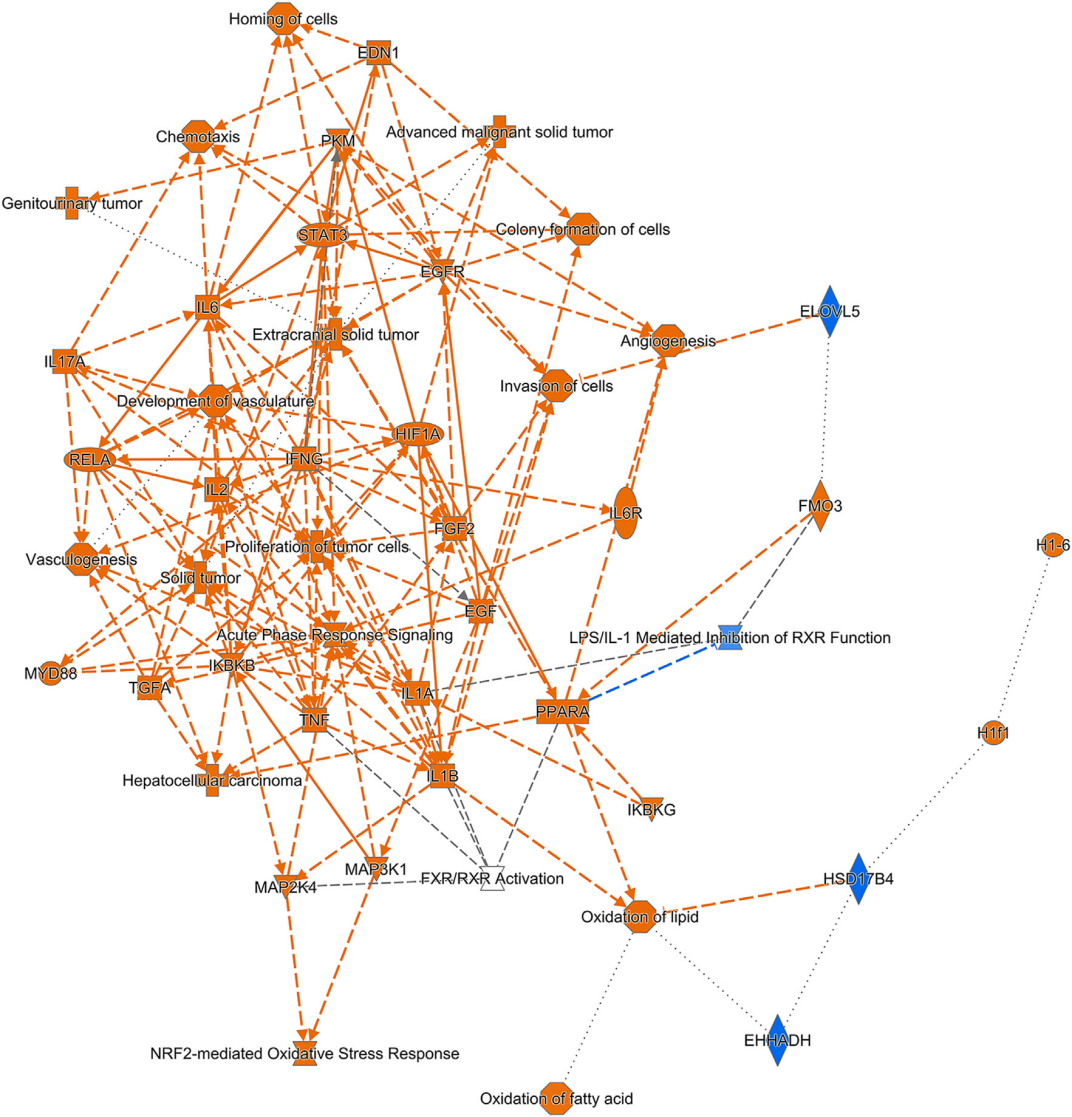
**Graphical summary showing activation or inhibition of key regulatory molecules, pathways, functions and phenotypes in Cluster one of AEG-1-C75S liver compared to AEG-1-WT.***Orange color* indicates activation z-score >2, *while blue color* indicates inhibition z-score <2. The *shapes* indicate the following: *octagon*: function; *cross*: disease; *inverted triangle*: kinase; *ellipse*: transcription regulator; *square*: cytokine; *trapezoid*: transporter; *circle*: molecule of other class; hour glass: canonical pathway.

**Supporting Figure S2:**
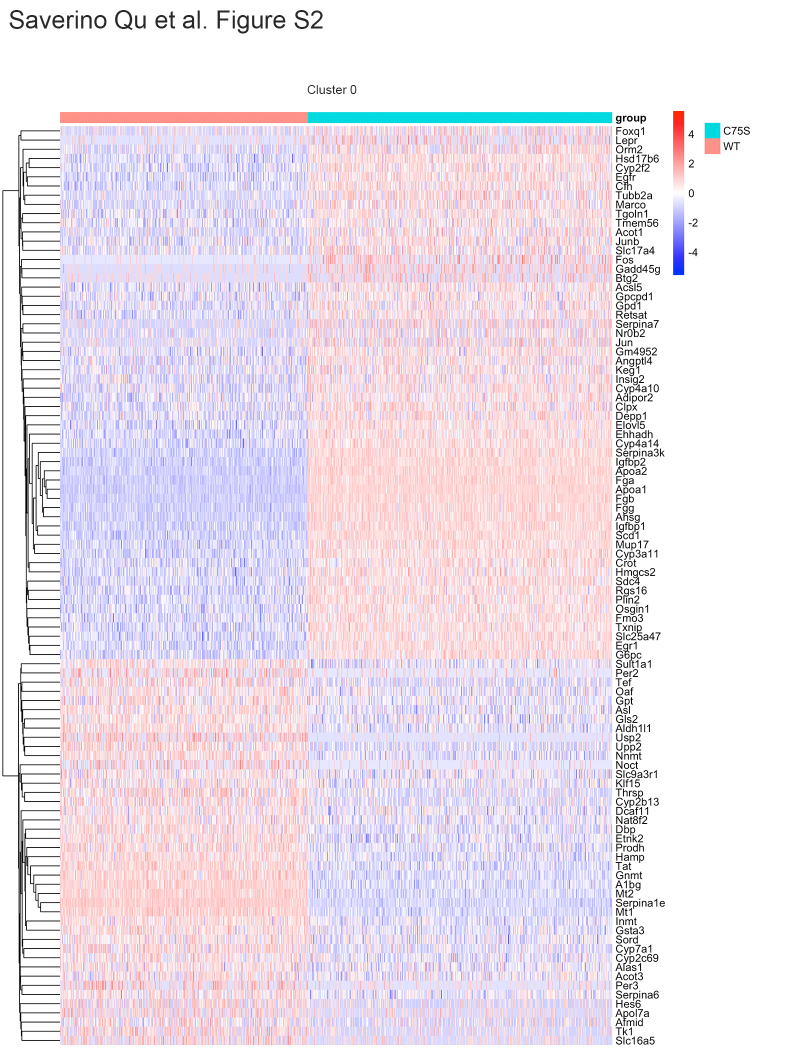


**Supporting Figure S3:**
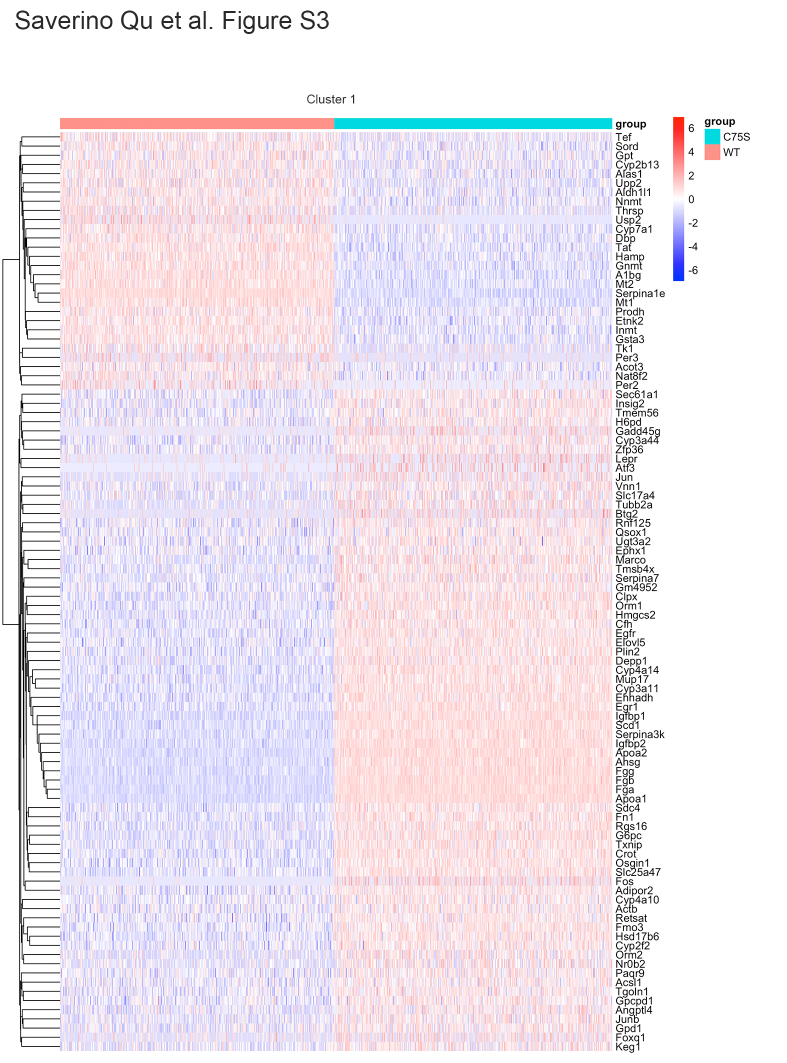


**Supporting Figure S4:**
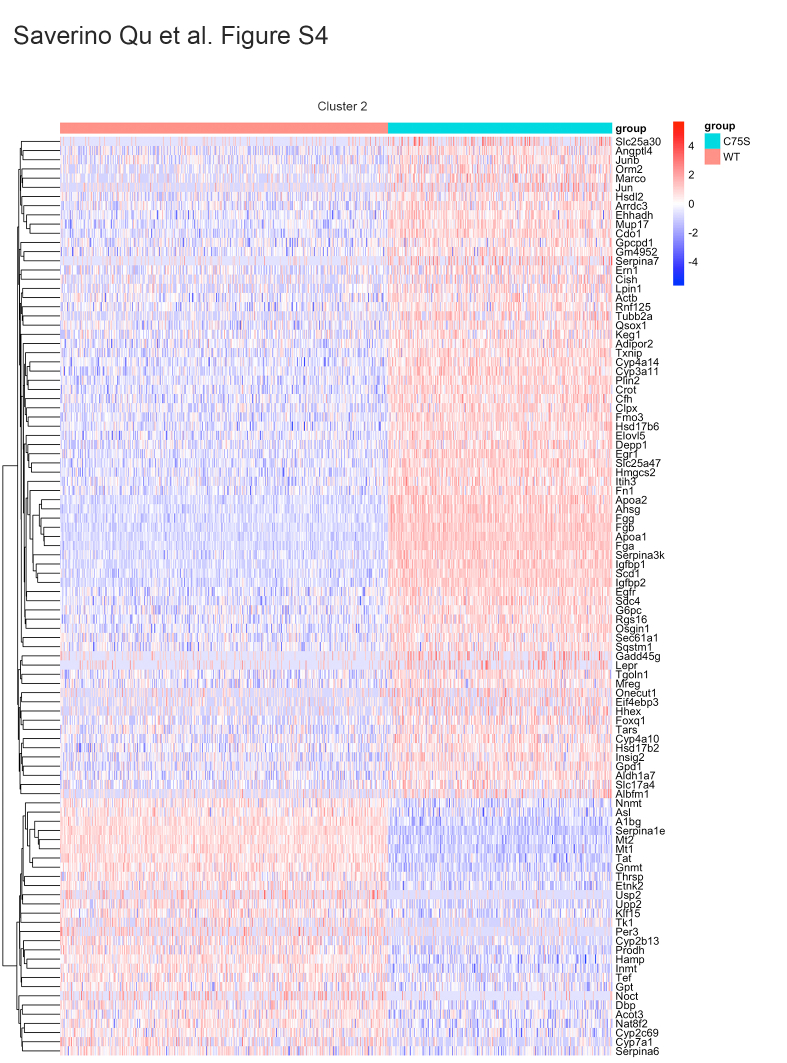


**Supporting Figure S5:**
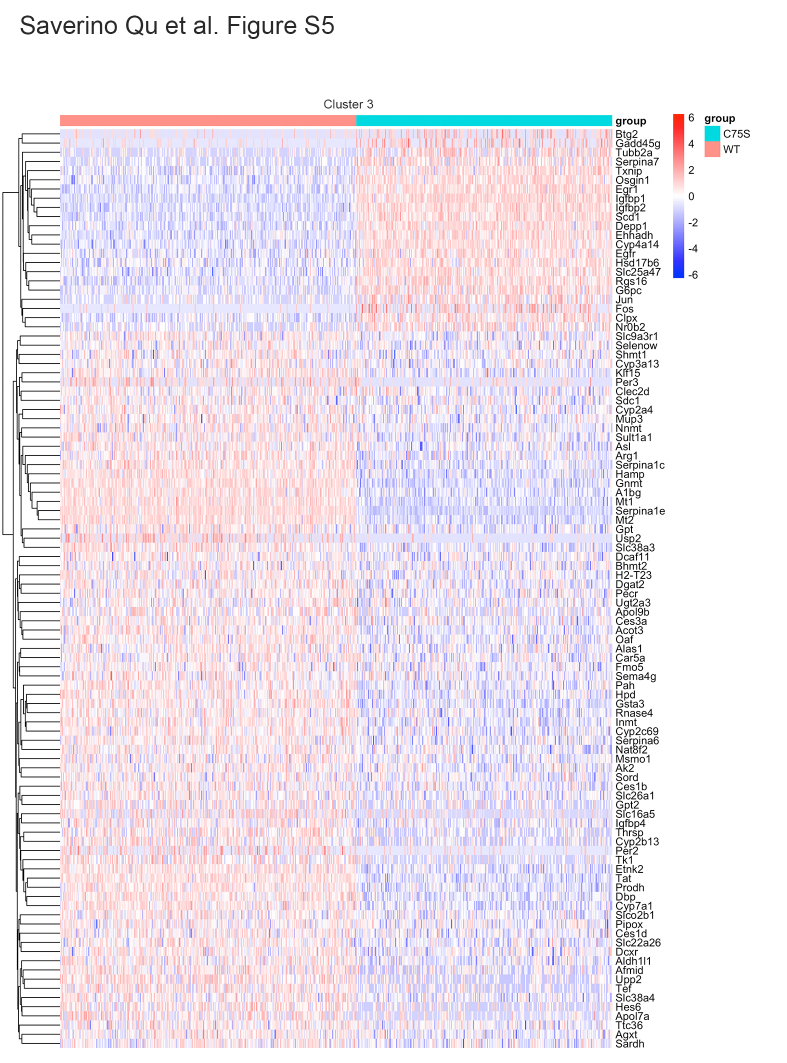


**Supporting Figure S6:**
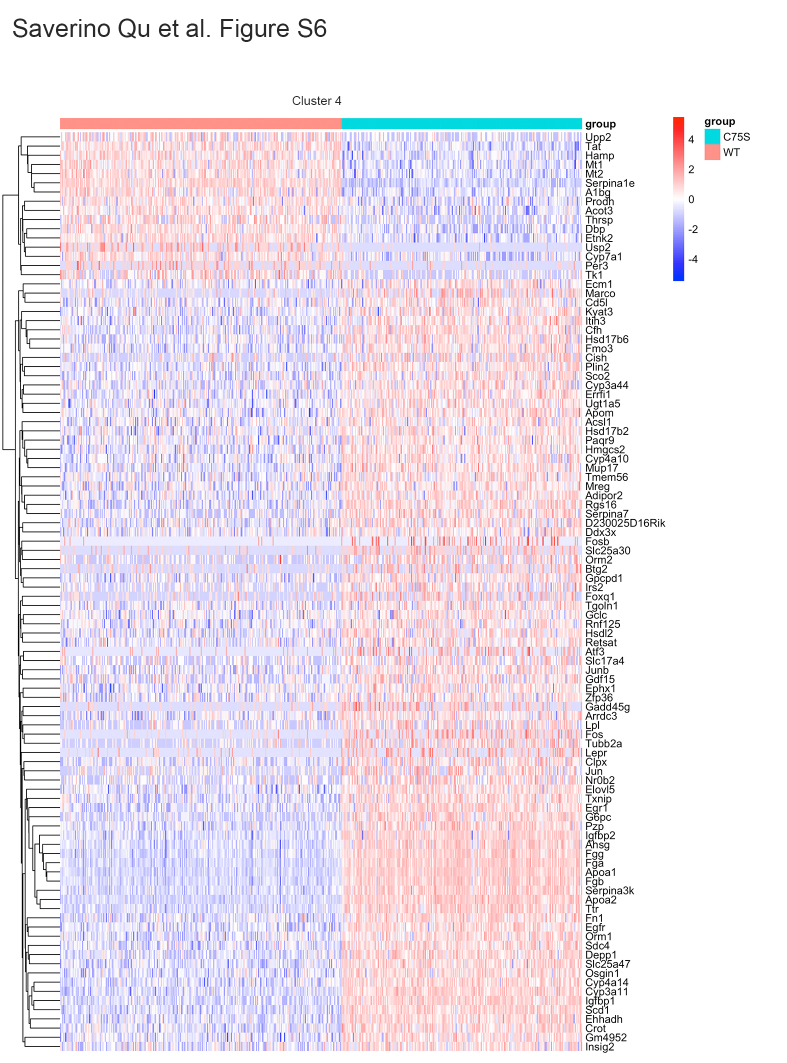


**Supporting Figure S7:**
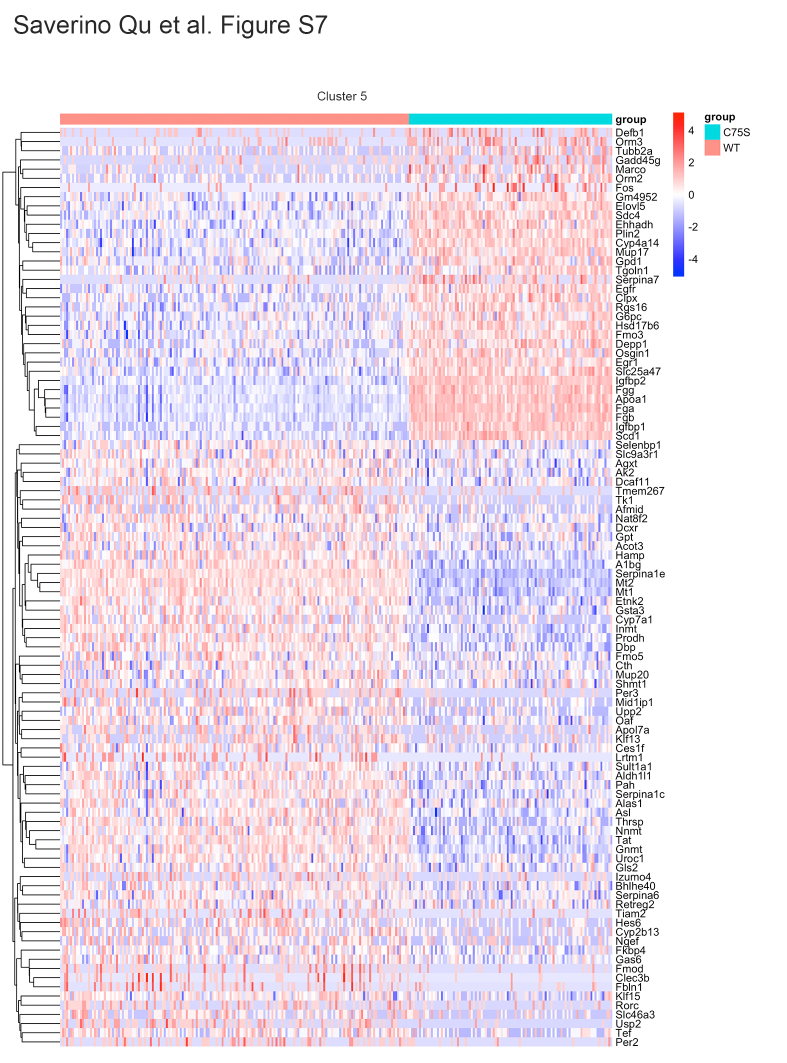


**Supporting Figure S8:**
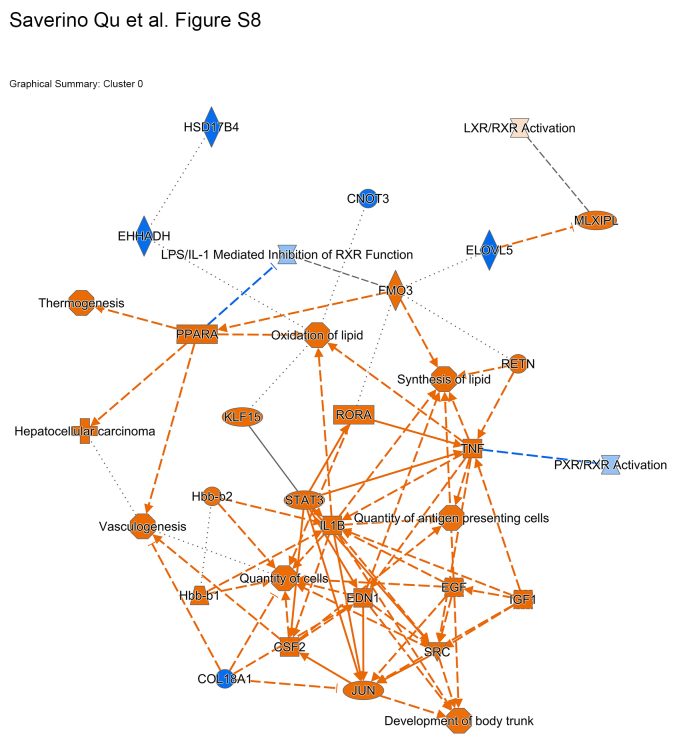


**Supporting Figure S9:**
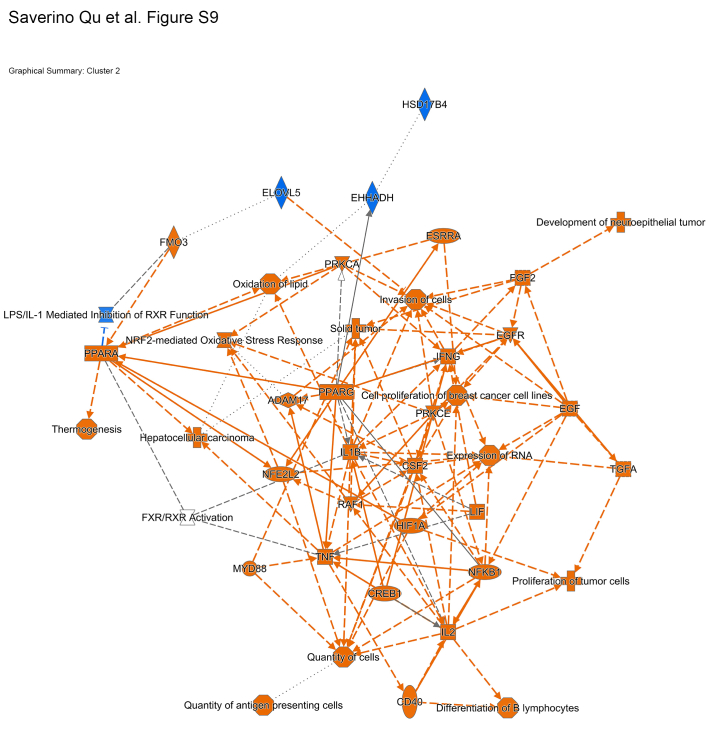


We did not observe any significant difference in the number of spatial spots in each of the six clusters between AEG-1-WT and AEG-1-C75S livers (data not shown). We, therefore, focused our analysis on differentially expressed genes (DEGs) in each cluster across the two samples. Using a cut-off of adjusted *p*-value 0.05, 350, 373, 334, 448, 398, and 87 genes were differentially expressed in clusters 0, 1, 2, 3, 4, and 5, respectively, in AEG-1-C75S liver compared to AEG-1-WT liver (Table S2 and Figs. S2–S7). The DEGs were subjected to Ingenuity Pathway Analysis (IPA) to determine canonical pathways and upstream regulators disrupted in each cluster, revealing significantly altered pathways resulting from the AEG-1-C75S mutation. In AEG-1-C75S, a positive z-score of two or more indicates activation of a pathway or upstream regulator, and a negative z-score of two or less indicates inhibition of a pathway or upstream regulator. Cluster five represented the least number of DEGs, and showed modulation of only three canonical pathways, suggesting that AEG-1-C75S mutation did not exert a significant effect on gene regulation in cholangiocytes. Clusters 0, 1, 2, and 4, representing periportal and pericentral hepatocytes, showed similar patterns of changes in canonical pathways (Figs. 6 and 7), which include activation of oncogenic signaling pathways (such as EGF, IGF-1, PDGF, and ILK), activation of macrophages (indicated by nitric oxide and reactive oxygen species production) and hepatic fibrosis, indicating that AEG-1-C75S mutation facilitates oncogenic functions of AEG-1. A graphical summary of changes in key regulatory molecules, pathways, and phenotypes for Cluster 1 is shown in Figure 8. The graphical summary for Clusters 0, 2, and 4 is similar to the representative summary shown for Cluster 1 (Figs. S8–S10). In AEG-1-C75S liver, activation of cell proliferation and invasion were detected which were associated with activation of oncogenic MAP2K4, MAP3K1, TGFA, NRF2, and STAT3 signaling ultimately contributing to hepatocellular carcinoma (Fig. 6). Activation of HIF1A and angiogenesis, and TNF, IL1A, IL1B, IL2, IL6, and IKBKB, IKBKG and RELA (components of pro-inflammatory NF-κB signaling pathway) contributing to inflammation, was observed in AEG-1-C75S liver *versus* AEG-1-WT (Fig. 8).

### Widespread inhibition of xenobiotic metabolism pathways in mid-lobular hepatocytes

As cluster three exhibited the greatest expression for mid-lobular zone markers, the respective IPA results for this cluster can be related to the molecular activity occurring in mid-lobular hepatocytes. A number of xenobiotic metabolism (XM) pathways display notable inhibition; these include PXR/RXR Activation, LXR/RXR Activation, XM CAR Signaling Pathway, XM PXR Signaling Pathway, and XM AHR Signaling Pathway (Fig. 6). Cytochrome P450 (CYP) genes play key roles in these processes, and isoforms belonging to CYP subfamilies 1 to 3 are responsible for the metabolism of nearly 80% of clinically-administered drugs (18). HCC tissues have also been reported to show a substantial decrease in CYP gene expression when compared to reference tissue, and HCC tumors with downregulated CYPs may be more susceptible to drug-induced liver toxicity (19). We observed downregulation of Cyp7a1, Cyp2a4, Cyp3a13, Cyp17a1, and Cyp1a2 in the mid-lobular zone of the C75S liver sample (Table S2), furthermore suggesting impaired XM function.

## Discussion

The goal of this project was to gain insight into the spatial organization of the liver and the potential impacts AEG-1 has on gene regulation. ST has emerged as a powerful tool for investigating the molecular and spatial complexity of tissues (20). This technology allowed us to observe and compare tissue-wide patterning of gene expression between livers of AEG-1-WT and AEG-1-C75S littermates. Overall, our analysis revealed distinct gene expression properties that differ based largely on liver zonation. These differences in the transcriptomic landscapes were further employed to generate biological insights related to metabolic activity in the presence of the C75S mutation.

ST first classified six distinct clusters based on shared patterns of gene expression, providing results that were central in unveiling the complex spatial organization of the liver. Importantly, our data strongly suggested a link between these clusters and the concept of liver zonation, and these results correspond with other studies investigating expression-driven zonation (21, 22). Not only does this information broaden our knowledge of the liver’s molecular landscape but also offers valuable insights into the potential functional implications of these distinct clusters. In the context of HCC, where alterations in liver architecture and expression profiles are often associated with disease progression (23), our findings may contribute to furthering the understanding of the underlying mechanisms of disease pathogenesis with increased specificity. Further investigations into the specific roles of these identified clusters in HCC may unveil novel therapeutic targets and treatment strategies.

The zone-specific marker analysis has enabled us to localize the periportal, mid-lobular, and pericentral zones within liver tissue. This localization is essential, as it unveils the unique patterns of expression within these zones in the context of MASH, HCC, and AEG-1-C75S mutation. The identification of clear zonation provides crucial information regarding the distribution of hepatocytes and neighboring populations, shedding light on the transcriptomic and metabolic properties correlating with certain hepatic regions. Interrogating the spatial context of these zones can be pivotal for uncovering the molecular heterogeneity associated with MASH and HCC development and progression. This insight may contribute to a more nuanced awareness of the spatial dynamics of differential gene expression in MASH and HCC, with the ability to detect potential biomarkers or therapeutic targets tailored to specific hepatic regions affected by this disease. These findings contribute to our understanding of the intricate zonal architecture of the liver and may have implications for MASH and HCC, where zonal alterations have been demonstrated throughout stages of MASH and HCC progression (24).

Targeted clustering analyses also yielded the localization of Kupffer cells, cholangiocytes, and hepatic stellate cell populations, providing an additional layer of specificity in characterizing the murine livers. Understanding the precise spatial arrangement of the three cell types is vital to unraveling the intricate cellular interactions within the MASH- and HCC-relevant liver microenvironment. This spatial information enhances our ability to discern the unique roles these subpopulations play in liver physiology and disease pathology, while also serving as a foundation for research efforts aimed at classifying any dysfunctions in specific cell types in disease states. Moreover, this notion holds relevance to AEG-1 such that changes in cellular behavior influenced by C75S mutations can be examined with increased accuracy.

The extensive inhibition of XM pathways in C75S mid-lobular hepatocytes carries significant implications for both our understanding of molecular alterations in this context and the potential clinical ramifications, especially in the context of HCC. IPA results displayed a distinct molecular profile in Cluster 3, marked by the upregulation of mid-lobular zone markers and a pronounced downregulation of key XM pathways. Cytochrome P450 (CYP) genes encode enzymes with unequivocal importance to the efficient processing and detoxification of xenobiotics, encompassing the paramount enzymatic system in Phase I drug metabolism (25). We observed the downregulation of several CYP genes in the mid-lobular zone of the C75S liver, further signifying compromised XM function. Cyp1a2, the primary cytochrome P450 enzyme present in the liver, plays a pivotal role in metabolizing about 8.9% of drugs commonly administered in clinical settings (26). It has been reported that HCC tissues show decreased Cyp1a2 expression when compared to non-tumor tissues (27, 28). Cluster three data exhibited Cyp1a2 downregulation, and IPA noted the involvement of Cyp1a2 across several of the inhibited XM pathways (*i.e.*, XM AHR Signaling, XM CAR Signaling, PXR/RXR Activation). Therefore, the underexpression of Cyp1a2 and other CYP genes detected in Cluster 3 coincides with the vast XM pathway inhibition in the mid-lobular zone. Retinoid X receptor α (RXRα), a nuclear receptor, forms heterodimers with key xenobiotic nuclear receptors, such as PXR and CAR, thereby activating transcription of target genes involved in XM. *In vivo* studies demonstrate RXRα in hepatocytes is vital to XM processes, evidenced by a significant drop in CYP gene expression in RXRα-deficient mice compared to WT (29). Interestingly, RXRα demonstrated notable inhibition in Cluster 3, and IPA further identified several CYP genes (Cyp1a2, 3a5, 4a11, 7a1) as targets of RXRα. We previously demonstrated that AEG-1 interacts with RXR and inhibits transcription co-activator recruitment thereby abrogating RXR-mediated gene transcription (30, 31). Inhibition of RXRα in AEG-1-C75S further indicates gain-of-function of this mutant compared to AEG-1-WT. Taken together, these observations collectively suggest dysregulated XM activity in the mid-lobular hepatocytes of the C75S liver.

These findings raise concerns regarding the potential susceptibility of HCC tumors, particularly those with AEG-1 mutations, to drug-induced liver toxicity. Drug-induced toxicity, MASH, and HCC all possess innate capabilities to induce liver damage. Upon these injuries, mid-lobular hepatocytes bear the chief responsibility for maintaining homeostasis *via* cellular repopulation compared to the periportal and pericentral hepatocytes (32). Due to dysregulated XM has seen in the C75S mid-lobular zone, it is possible that these hepatocytes are less efficient at fulfilling their role in maintaining liver homeostasis. Administering drugs in a state of reduced XM function and CYP expression may lead to hepatic toxicity and homeostatic imbalances, potentially furthering the pathogenic progression of HCC. Understanding these alterations is crucial for advancing our knowledge of HCC pathogenesis and may inform future therapeutic strategies tailored to the distinct molecular characteristics of mid-lobular hepatocytes in the context of AEG-1 mutations.

In this study, ST proved instrumental in depicting the elaborate molecular landscape of liver tissue, shedding light on its spatial organization and the impacts of the AEG-1 mutation in HCC. Our research identified distinct cellular clusters, revealing the complex spatial composition of the liver and emphasizing the relevance of liver zonation in determining spatial dynamics of metabolic functions. Moreover, targeted clustering analyses of cell type-specific markers outlined the distribution patterns of Kupffer cells, cholangiocytes, and hepatic stellate cells, offering nuanced insights into the cellular architecture of the liver. Notably, the inhibition of xenobiotic metabolism pathways, particularly in mid-lobular hepatocytes with the C75S mutation, suggests compromised drug metabolism in HCC, with potential implications for increased susceptibility to drug-induced toxicity and disease progression. These findings not only expand our grasp on liver biology but also provide specific insights into the molecular intricacies of HCC, perhaps aiding the development of targeted therapies and personalized treatment strategies. The spatial resolution provided by ST proved indispensable in localizing distinct cell types and expression patterns, enhancing the precision of our findings and their relevance to HCC and AEG-1 mutations.

## Experimental procedures

### Mouse

The generation of AEG-1-C75S mouse by CRISPR/Cas9 technology has been described (16). AEG-1-C75S heterozygote x heterozygote mating was performed to generate AEG-1-WT and AEG-1-C75S littermates. All animal studies were approved by the Institutional Animal Care and Use Committee at Virginia Commonwealth University.

### Spatial transcriptomics

#### Tissue collection, preparation, and slide mounting

Mouse liver tissue (≤6.5 × 6.5 mm) was collected from identical lobes of 8-week-old naïve female WT and C75S littermates and fixed in 10% neutral buffered formalin for approximately 24 h. After paraffin embedding, RNA quality assessment (DV_200_) and tissue adhesion test were performed according to 10X Genomics protocol CG000408 (Rev A).

#### RNA quality assessment

Four 10 μm curls per sample were placed in pre-chilled 1.7 ml microcentrifuge tubes. RNA extraction was performed using the RNeasy FFPE kit (Qiagen, 73504) following the manufacturer’s protocol, and RNA concentration of the samples was measured using a Nanodrop One. The percentage of RNA fragments >200 nucleotides (DV_200_) was evaluated using the RNA 6000 Pico Kit (Agilent, 5067-1513) and the Agilent 2100 Bioanalyzer system (2100 Expert software version B.02.11.SI824).

#### Tissue adhesion test

Four 5 μm sections from each experimental tissue block were placed within the etched capture areas of a Visium Tissue Section Test Slide (10X Genomics, 1000347), dried for 3 h using a Thermocycler Adaptor (10X Genomics, 3000380) on a thermal cycler set to 42 °C, and stored in a desiccator overnight at room temperature. Deparaffinization and H&E staining were performed following 10X Genomics protocol CG000409, Rev C (see next paragraph for details), and the slide was examined for signs of tissue detachment. H&E-stained slides are shown in Fig. S1.

### Deparaffinization, H&E staining, imaging, and decrosslinking

Once confirmed that DV_200_ was >50% and tissue remained adhered to the slide throughout the staining process, one 5 μm section per sample was placed within the fiducial framed capture areas of a Visium Spatial Gene Expression Slide (10X Genomics, 2000233) following placement protocol above and stored in a desiccator. A single capture area of the Spatial Gene Expression slide contains 5000 gene expression spots, each with unique barcoded capture primer sequences.

#### Deparaffinization and H&E staining

The slide was incubated for 2 h using a Thermocycler Adaptor on a thermal cycler set to 60 °C. Once the slide cooled to room temperature, paraffin was removed with two changes of xylene. Sections were then rehydrated and H&E stained using Mayer’s hematoxylin (Millipore Sigma, 51275), Bluing Buffer (Fisher Scientific, 6769001), and alcoholic Eosin (Millipore Sigma, HT110116). After a short rinse in Milli-Q water, 85% glycerol and a glass coverslip were applied to the tissue sections. Imaging of the slide was conducted immediately after cover-slipping.

#### Imaging

Bright-field images of the tissue sections were acquired at 10X magnification using a Zeiss Axio Imager.Z2 microscope and Neurolucida software (MBF Bioscience). Each capture area on the slide was imaged individually, with the fiducial frames in focus to allow for successful alignment in SpaceRanger software. Immediately following the imaging, the coverslip was gently removed by holding the slide horizontally in Milli-Q water until the coverslip detached completely from the slide. The slide was dried briefly using a Thermocycler Adaptor on a thermal cycler set to 37 °C.

#### Decrosslinking

The slide was inserted into a Visium cassette with a gasket (10X Genomics, 2000281) to allow for the addition of reagents directly to individual tissue sections. The sections were washed three times with 0.1 N HCl and once with TE buffer (pH 9.0) before fresh TE buffer was added and the slide incubated for 1 h using a Thermocycler Adaptor on a thermal cycler set to 70 °C. For all of the above steps, 10X Genomics protocol CG000409 (Rev C) was followed.

### Probe hybridization/ligation/release/extension

We utilized the Visium Spatial for FFPE Gene Expression Kit, Mouse Transcriptome (PN-1000339, 10X Genomics), and User Guide CG000407, Rev D for the following steps.

#### Probe hybridization

TE buffer was removed from the wells and a pre-hybridization mixture containing a permeabilization enzyme and Tween-20 was added to the wells. After a 15-min incubation at room temperature, the solution was removed and a probe hybridization mix containing probe pairs specific to the mouse whole transcriptome was added to the wells. The cassette was sealed and the slide incubated overnight using a Thermocycler Adaptor on a thermal cycler set to 50 °C. Once hybridization was completed, the solution was removed from the wells and sections were washed three times with pre-heated (50 °C) SSC buffer diluted to 2X (S6639-1L, Millipore Sigma).

#### Probe ligation, release, and extension

Once the slide cooled to room temperature, buffer was removed from the wells and a ligation mixture added to the wells to ligate the probe pairs bound to the target RNA within the sections. The cassette was sealed and the slide incubated for 1 h at 37 °C in a thermal cycler. Once ligation was complete, the solution was removed from the wells, and sections were washed two times with a post-ligation wash buffer using a Thermocycler Adaptor on a thermal cycler set to 57 °C. This was followed by two changes of 2X SSC buffer. The slides were sealed and stored at 4 °C overnight. After removal of the wash buffer, the slide was incubated at 37 °C with an RNase mixture, followed by a permeabilization mixture, in order to release the ligated products from the cells and allow binding to the barcoded capture primers *via* a 30 nt poly(dT) sequence within the gene expression spots. The wells were washed with 2X SSC and an extension reaction was initiated (15 min at 45 °C) to add a 12 nt Unique Molecular Identifier (UMI) sequence, 16 nt spatial barcodes specific to the originating gene expression spot on the slide, and partial read one sequence (Illumina TruSeq Read 1) to the ligated product. After a wash with 2X SSC, the barcoded ligation product was eluted with 0.08 M KOH. 1M Tris-HCl pH 7.0 was added to the solution to stabilize pH, and the samples were stored at −20 °C overnight.

### Library construction, sequencing, and bioinformatics analysis

#### Library construction

To determine the correct cycle number for the sample index PCR, we ran a qPCR with the samples on a Quantstudio three using KAPA SYBR FAST qPCR master mix/ROX Low dye (Roche, KK4600) and TS Primer Mix A (10X Genomics, 2000447) following manufacturer’s instructions. Cq value for each sample was determined. The remainder of the samples were combined with an amplification mix (10X Genomics) and unique primer index sets (i7, i5) from two different wells of a Dual Index TS Set A plate (10X Genomics, 3000511). The sample index PCR program was run using Cq+2 for the number of cycles. SPRIselect reagent (Beckman Coulter, B23317) was used at a ratio of 0.85X to separate small fragments of DNA from the library. The samples were run at 1:5 dilution on an Agilent Bioanalyzer High Sensitivity chip to determine insert size for library quantification.

#### Sequencing

Libraries were pooled, loaded into a P2 flow cell, and sequenced using the Illumina NextSeq 2000 system following the paired-end dual-indexed sequencing configuration described in User Guide CG000407, Rev D.

#### Bioinformatics analysis

Raw reads obtained from the 10x Visium library were processed using Space Ranger v2.0.1. This software aligned the sequencing reads to the Gex-mm10 to 2020-A reference genome and probe set to generate gene-count matrices. Additionally, it quantified gene expression at each spatial location and integrated the data onto the corresponding tissue image, resulting in over 2000 spatial spots per tissue sample. Filtered feature-barcode matrices and spatial coordinates were imported into R using the Seurat package (v4.3.0) (33). Spot outliers with read counts exceeding 50,000 or feature numbers below 100 or greater than 7000 were removed. Subsequently, SCTransformation was applied to normalize gene expression data, utilizing regularized negative binomial regression to eliminate technical variation while preserving biological variance. Samples were then merged into a single object for joint dimensional reduction and clustering, reducing the dimensionality to 50 principal components using PCA. A Shared Nearest Neighbor (SNN) approach was employed to construct a graph representation, followed by the Louvain algorithm to detect clusters of spatial spots. The clustering results were visualized using UMAP and spatial plots. To identify predominant cell types across spatially resolved regions and distinct hepatic zones, all spots were reclustered using a curated selection of gene markers known for their high specificity in distinguishing between different cell types and zonation patterns within liver tissue. Specifically focusing on Kupffer cells, cholangiocytes, and stellate cells, we examined the expression profiles of 11 selected cell type-specific markers for clustering analysis, achieving a higher resolution to delineate distinct clusters corresponding to the targeted cell types. For zonation analysis, a similar approach was adopted, clustering spots based on the expression profiles of 12 zone-specific markers, albeit with a lower resolution whereby 3 clusters were identified corresponding to established liver zones. Differentially expressed genes were analyzed for canonical pathways and upstream regulators using Ingenuity Pathway Analysis (IPA) using the built-in analysis software.

## Data availability

The ST data is available on GEO under GSE261057 accession number. All other data are contained within the manuscript.

## Supporting information

This article contains supporting information.

## Conflict of interest

The authors declare that they have no known competing financial interests or personal relationships that could have appeared to influence the work reported in this paper.

## Figures and Tables

**Supporting Figure S10:**