# Supplementary material for: Spatial transcriptomics unravels palmitoylation and zonation-dependent gene regulation by AEG-1 in mouse liver
Source: J Biol Chem. 2024 Apr 25;300(6):107322. doi: 10.1016/j.jbc.2024.107322 (PMC11134871; doi:10.1016/j.jbc.2024.107322)
Supplement: Supporting Information [file mmc3.docx]

**Supplementary Figure and Table Legends**

Figure S1. H&E-stained images of the slides of AEG-1-WT and AEG-1-C75S livers subjected to ST analysis.

Figure S2. Heat map of top 100 differentially expressed genes in cluster 0 of AEG-1-WT and AEG-1-C75S livers identified by ST analysis. Each bar represents one spatial spot.

Figure S3. Heat map of top 100 differentially expressed genes in cluster 1 of AEG-1-WT and AEG-1-C75S livers identified by ST analysis. Each bar represents one spatial spot.

Figure S4. Heat map of top 100 differentially expressed genes in cluster 2 of AEG-1-WT and AEG-1-C75S livers identified by ST analysis. Each bar represents one spatial spot.

Figure S5. Heat map of top 100 differentially expressed genes in cluster 3 of AEG-1-WT and AEG-1-C75S livers identified by ST analysis. Each bar represents one spatial spot.

Figure S6. Heat map of top 100 differentially expressed genes in cluster 4 of AEG-1-WT and AEG-1-C75S livers identified by ST analysis. Each bar represents one spatial spot.

Figure S7. Heat map of top 100 differentially expressed genes in cluster 5 of AEG-1-WT and AEG-1-C75S livers identified by ST analysis. Each bar represents one spatial spot.

Figure S8. Graphical summary showing activation or inhibition of key regulatory molecules, pathways, functions and phenotypes in Cluster 0 of AEG-1-C75S liver compared to AEG-1-WT. Orange color indicates activation z-score >2, while blue color indicates inhibition z-score <2. The shapes indicate the following: octagon: function; cross: disease; inverted triangle: kinase; ellipse: transcription regulator; square: cytokine; trapezoid: transporter; circle: molecule of other class; hour glass: canonical pathway.

Figure S9. Graphical summary showing activation or inhibition of key regulatory molecules, pathways, functions and phenotypes in Cluster 2 of AEG-1-C75S liver compared to AEG-1-WT. Orange color indicates activation z-score >2, while blue color indicates inhibition z-score <2. The shapes indicate the following: octagon: function; cross: disease; inverted triangle: kinase; ellipse: transcription regulator; square: cytokine; trapezoid: transporter; circle: molecule of other class; hour glass: canonical pathway.

Figure S10. Graphical summary showing activation or inhibition of key regulatory molecules, pathways, functions and phenotypes in Cluster 4 of AEG-1-C75S liver compared to AEG-1-WT. Orange color indicates activation z-score >2, while blue color indicates inhibition z-score <2. The shapes indicate the following: octagon: function; cross: disease; inverted triangle: kinase; ellipse: transcription regulator; square: cytokine; trapezoid: transporter; circle: molecule of other class; hour glass: canonical pathway.

Table S1. List of highly expressed genes in each cluster.

Table S2. List of differentially expressed genes in each cluster. Log2FC indicates Log2 fold change in gene expression in AEG-1-C75S liver versus AEG-1-WT.
